# Exercise, CaMKII, and type 2 diabetes

**DOI:** 10.17179/excli2020-3317

**Published:** 2021-02-17

**Authors:** Jitcy S. Joseph, Krishnan Anand, Sibusiso T. Malindisa, Adewale O. Oladipo, Oladapo F. Fagbohun

**Affiliations:** 1 Department of Toxicology and Biochemistry, National Institute for Occupational Health, A division of National Health Laboratory Service, Johannesburg, South Africa; 2 Department of Chemical Pathology, School of Pathology, Faculty of Health Sciences and National Health Laboratory Service, University of the Free State, Bloemfontein, South Africa; 3 Department of Life and Consumer Sciences, University of South Africa (UNISA), Florida Park, Johannesburg, South Africa; 4 Institute for Nanotechnology and Water Sustainability (iNanoWS), College of Science, Engineering and Technology, University of South Africa, Science Park Florida, Johannesburg, 1710, South Africa; 5 Department of Biomedical Engineering, First Technical University, Ibadan, Oyo State, Nigeria; 6 Department of Pediatrics, Group on the Molecular and Cell Biology of Lipids, University of Alberta, Edmonton, AB, Canada

**Keywords:** exercise, Type 2 diabetes, CaMKII, GLUT4, mitochondrial biogenesis, insulin resistance

## Abstract

Individuals who exercise regularly are protected from type 2 diabetes and other metabolic syndromes, in part by enhanced gene transcription and induction of many signaling pathways crucial in correcting impaired metabolic pathways associated with a sedentary lifestyle. Exercise activates Calmodulin-dependent protein kinase (CaMK)II, resulting in increased mitochondrial oxidative capacity and glucose transport. CaMKII regulates many health beneficial cellular functions in individuals who exercise compared with those who do not exercise. The role of exercise in the regulation of carbohydrate, lipid metabolism, and insulin signaling pathways are explained at the onset. Followed by the role of exercise in the regulation of glucose transporter (GLUT)4 expression and mitochondrial biogenesis are explained. Next, the main functions of Calmodulin-dependent protein kinase and the mechanism to activate it are illustrated, finally, an overview of the role of CaMKII in regulating GLUT4 expression, mitochondrial biogenesis, and histone modification are discussed.

## Introduction

Type 2 diabetes is one of the fast moving public health problems in both developed and developing countries. According to the World Health Organization (WHO), developing countries will likely suffer from diabetes epidemics in the 21^st^ century. In developing countries, around 50 % of people with diabetes are undiagnosed. As a result, many do not receive adequate treatment and care to manage the disease, putting them at greater risk of serious complications and even death. According to the International Diabetes Federation (IDF) (Atlas ninth edition, 2019), the number of people with type 2 diabetes is increasing in every country. In 2019, diabetes affected at least 463 million people worldwide and is expected to reach 700 million by 2045 (IDF). The economic impact of diabetes is expected to continue to grow by 2045 as there is no effective cure to prevent or treat diabetes.

Regulation of carbohydrate and lipid metabolism is very important in patients with insulin resistance and type 2 diabetes. Glucose is most commonly utilized for energy production in mammals. The regulation of glucose metabolism is very important to ensure glucose availability for the central nervous system, which almost entirely depends on glucose for its fuel. Under hyperglycemic conditions, glycogen synthesis is the major pathway in glucose metabolism, and muscle glycogen synthesis rate decreases by 50 % in type 2 diabetes as compared with healthy subjects (Shulman et al., 1990[95]). Skeletal muscle is responsible for > 80 % of whole-body glucose disposal, which is primarily facilitated by glucose transporter protein (GLUT)4 (Shulman et al., 1990[95]). Insulin-mediated glucose uptake, oxidation, and storage by the skeletal muscle are severely impaired in type 2 diabetes (Kelley et al., 2002[46]). This is due to increased numbers of intramyocellular lipid species such as ceramides and diacylglycerol that interfere with GLUT4 translocation through the inhibition of the insulin receptor substrate (IRS) (Kraegen and Cooney, 2008[52]). IRS proteins are involved to recruit phosphatidylinositol-3-kinase (PI3K) in the insulin-signaling pathway, which in turn phosphorylates and activates the serine/threonine kinase Akt (Protein kinase B). Akt is a serine/threonine kinase, which is involved in mediating several biological responses, like inhibition of apoptosis and stimulation of cell proliferation. Activation of Akt then leads to the translocation of intracellular vesicles containing GLUT4 to fuse with the plasma membrane, which results in glucose uptake in the cell (Rowland et al., 2011[92]).

Impairment of insulin-mediated glucose uptake correlates with altered fatty acid metabolism as the biochemical pathways of fatty acid and glucose metabolism are fully integrated (Randle, 1998[85]). Lipids are a fuel source used for the production of energy. Elevated free fatty acids can cause insulin resistance that leads to an increase in the blood glucose level, which is the hallmark of type 2 diabetes. Increased free fatty acids levels disturb the insulin-signaling pathway, ultimately reducing the translocation of glucose transporter molecules to the plasma membrane and decreasing cellular glucose uptake (McGarry et al., 1983[67]). Energy intake exceeding its expenditure usually causes defects in fatty acid metabolism (Groop et al., 1991[28]). Physical inactivity also contributes to an imbalance between fatty acid oxidation and supply. Decreased fatty acid oxidation leads to an accumulation of fatty acids and their intermediates (diacylglycerol and ceramides), which disrupts the insulin-stimulated pathway of glucose uptake in skeletal muscle (Roden et al., 1996[89]; Kelley and Mandarino, 2000[47]). 

Calcium (Ca^2+^) is an important second messenger involved in the regulation of many cellular events (Berridge et al., 2000[6]). Raising cytosolic calcium levels in muscle activates calcium/calmodulin-dependent protein kinase (CaMK), a class of multifunctional serine/ threonine-specific protein kinase. CaMKII is the dominant isoform of CaMK in human skeletal muscle (Rose et al., 2006[91]). It is reported that exercise activates CaMKII and results in both mitochondrial biogenesis and improved glucose transport in parallel (Wright et al., 2007[106]; Raney and Turcotte, 2008[86]; Wu et al., 1999[107]). This parallel up-regulation has got positive implications in alleviating type 2 diabetes symptoms. This review will discuss the following: 1) Role of exercise in regulating insulin-signaling pathway, lipid metabolism, carbohydrate metabolism, GLUT4 expression and mitochondrial biogenesis; 2) Mechanism of Calmodulin-dependent protein kinase (CaMK)II activation in skeletal muscle; 3) Role of CaMKII in GLUT4 expression, mitochondrial biogenesis, and histone modification.

### Exercise regulates the insulin-signaling pathway, lipid metabolism, and carbo-hydrate metabolism

Exercise or physical activity is one of the cornerstones for the prevention and management of type 2 diabetes in both men and women (ADA, 2004[1]). Several studies and clinical trials have assessed the role of exercise or physical activity on type 2 diabetes. A study by Tuomilehto et al. (2001[100]) shows that modest weight loss of 5-7 % could be achieved by moderate exercise of at least 30 min per day (150 min per week) resulting in lowering the possibility of developing diabetes by 58 % in overweight people with pre-diabetes. In people with type 2 diabetes, exercise improves glycemic control and reduces the risk of cardiovascular diseases and total mortality (Hu et al., 2005[41]; Dunstan et al., 2005[18]). Moderate-intensity exercise increases plasma glucose oxidation and decreases glycogen oxidation in type 2 diabetes as compared with healthy subjects. The protective effects of exercise were strongest in persons with the highest risk of type 2 diabetes, that is people with a high body mass index, a history of hypertension, or a family history of diabetes. 

Regular exercise also reduces the risk of ectopic (non-adipose tissue) lipid accumulation and type 2 diabetes through increased lipid oxidation and lipid oxidative capacity (Martin et al., 1995[65]). A study by Blaak et al. (2000[7]) reported that exercise decreases oxidation of plasma free fatty acids and increases oxidation of triglycerides-derived fatty acids in type 2 diabetes subjects as compared with control subjects. Furthermore, exercise increases the oxidation of intramuscular triglycerides and reduces lipid intermediates in type 2 diabetes patients. Reducing the circulation of fatty acids is an effective strategy to improve insulin sensitivity in type 2 diabetes. These studies indicate that exercise positively affects glucose and fatty acid uptake, supply, and oxidation.

With regards to insulin sensitivity, exercise also shows a beneficial effect on insulin sensitivity in normal as well as insulin-resistant populations. Individuals with insulin resistance and type 2 diabetes are characterized by impaired insulin-stimulated glucose uptake in skeletal muscle (Zierath et al., 1996[108]). However, acute exercise increases glucose uptake in skeletal muscle via an insulin-independent mechanism that bypasses the insulin signaling defects associated with these conditions (Wallberg-Henriksson and Holloszy, 1985[102]; Cortez et al., 1991[12]; Zierath et al., 2000[109]; Christ-Roberts et al., 2004[9]; O'Gorman et al., 2006[80]). Studies show that aerobic exercise increases insulin signaling and glucose transporter content in skeletal muscle (Sriwijitkamol et al, 2007[97]; O'Gorman et al., 2006[80]). It is reported that a single bout of exercise increases insulin sensitivity for at least 16 hours' post-exercise in healthy as well as in type 2 diabetes subjects. In an experiment with 14 obese patients with type 2 diabetes, Kirwan et al. (2009[50]) reported that one week of vigorous exercise induces significant improvements in insulin action in type 2 diabetes. These improvements involve elevated peripheral insulin sensitivity and responsiveness as well as increased suppression of hepatic glucose production. A study by Araki et al. (1994[2]) shows that ten weeks of endurance training increased insulin sensitivity in rats. Exercise restores the level of insulin sensitivity to normal in diabetic patients (Cortez et al., 1991[12]). Collectively, these studies show that exercise improves insulin sensitivity, regulates lipid and carbohydrate metabolism, and reduces the risk of type 2 diabetes.

### Exercise improves GLUT4 expression and protects from type 2 diabetes

Reduction in the risk of diabetes by exercise is owing to increased glucose transport capacity (Deisseroth et al., 1998[15]; Lakka and Tuomilehto, 2007[60]). Regular exercise induces the capacity of skeletal muscle to oxidize pyruvate and fatty acids and take up glucose (Ren et al., 1994[87]; Holloszy and Booth, 1976[37]; Lakka and Tuomilehto, 2007[60]). This can prevent and even reverse type 2 diabetes. The increase in glucose uptake capacity is due to the increase in the content of glucose transporter protein (GLUT)4 (Ren et al., 1994[87]). Skeletal muscle is the major tissue responsible for insulin-mediated glucose utilization (Baron et al., 1988[4]) and greatly contributes to the postprandial hyperglycemia observed in individuals with type 2 diabetes (DeFronzo et al., 1982[14]; Dohm et al., 1988[16]). Several studies reported that insulin-mediated glucose uptake and utilization is considerably weakened in type 2 diabetic patients skeletal muscle (Eriksson et al.,1992[20]; Shulman et al., 1990[95]; DeFronzo et al., 1985[13]).

Glucose transport is significant for glucose utilization under most physiologic conditions in skeletal muscle (Kubo and Foley, 1986[57]). Glucose is transported into cells through the plasma membrane and T-tubules via facilitated transport using glucose transporter proteins. The main function of the GLUT4 protein is to facilitate glucose uptake into cells and maintain control of blood glucose levels. GLUT4 protein in the basal state is stored in intracellular vesicles and their translocation to the plasma membrane mainly occurs by insulin action or through insulin-independent pathways during muscle contraction. Insulin resistance may result from impaired insulin signal transduction, leading to decreased GLUT4 translocation and/or diminished capacity for GLUT4 synthesis (Garvey et al., 1998[24]). Exercise increases insulin-stimulated glucose disposal and GLUT4 protein content in obese patients with type 2 diabetes (O'Gorman et al., 2006[80]).

**Figure 1:**
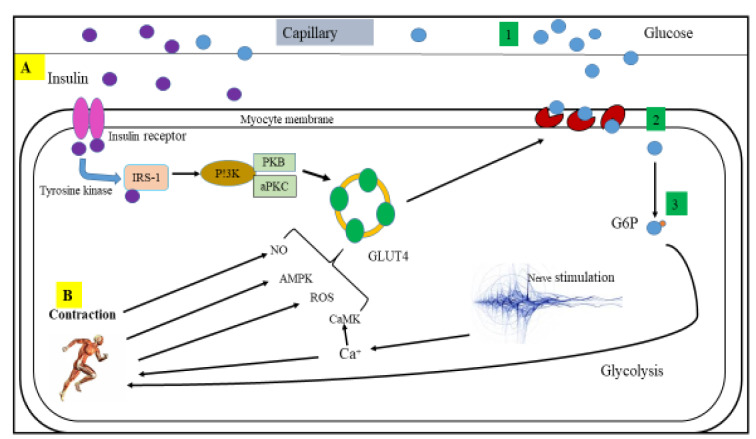
Mechanisms of glucose uptake into skeletal muscle: (A) Insulin-activated glucose uptake, (B) Possible mechanisms associated in contraction-stimulated glucose uptake; 1) glucose transportation to the muscle cell, 2) glucose delivery via the membrane, and 3) glucose phosphorylation and then flux through metabolism. CaMK, calmodulin-dependent protein kinase; aPKC, atypical protein kinase C; ROS, reactive oxygen species; AMPK, AMP-activated protein kinase; IRS-1, insulin receptor substrate 1 PI3K, phosphoinositide-3 kinase; G6P, glucose-6-phosphate; NO, nitric oxide and PKB, protein kinase B/Akt

Plasma membrane GLUT4 content correlated with glucose transport activity in both rat and human skeletal muscle (Lund et al., 1998[64]). A study by Kennedy et al. (1999[48]) shows that plasma membrane GLUT4 increases by 70 % above the basal level in skeletal muscle from healthy and type 2 diabetic patients during acute cycle exercise. A single exercise bout also resulted in elevated skeletal muscle GLUT4 mRNA immediately after exercise in human skeletal muscle (Kraniou et al., 2006[54], 2004[55]; McGee and Hargreaves, 2004[69]). It is also reported that exercise increases plasma membrane GLUT4 content and glucose transport in insulin-resistant obese Zucker rats (Hansen et al., 1998[32]). These studies show that exercise increases plasma membrane GLUT4 in both animals and humans. Moreover, exercise effectively translocate GLUT4 to the cell surface and increases glucose transport in insulin-resistant and diabetic subjects. Studies also show that exercise-induced GLUT4 translocation is mediated by insulin signaling pathways, which is different from that of being induced by insulin (Lund et al., 1998[64]; Goodyear et al., 1995[26]; Coderre et al., 1995[10]), indicating that insulin and exercise use different pools of GLUT4 transporters. The mechanisms through which exercise stimulates GLUT4 translocation and glucose uptake appear to arise from local factors within skeletal muscle such as calcium, calmodulin-dependent protein kinase, reactive oxygen species (ROS), nitric oxide (NO), and AMP-activated protein kinase (AMPK) as shown in Figure 1(Fig. 1) (Merry and McConell, 2009[74]).

Several studies show that exercise increases glucose transport and GLUT4 expression in skeletal muscle. A vigorous 7-day exercise program increased insulin sensitivity and muscle GLUT4 content in younger and older people (Kirwin et al., 2003[51]). Again, a study done by Kim et al. (1999[49]) determined the effects of exercise on adaptations of skeletal muscle, including GLUT4 proteins and intra-muscular triglyceride concentration (IMTG). Non-obese elderly Korean men (age range 58-67 years) with impaired glucose tolerance performed 12 weeks of endurance exercise (60-70 % of the heart rate reserve). Exercise improved total GLUT4 protein expression, decreased IMTG and increased fatty acid oxidation capacity. The effects of exercise on adipose tissue and skeletal muscle GLUT4 protein expression were investigated in patients with type 2 diabetes. Muscle and adipose tissue samples were collected before and after 4 weeks of exercise in 7 patients with diabetes [47 ± 2 years). Seven control subjects were used for baseline comparison. Adipose tissue GLUT4 protein expression was 43 % lower in patients with diabetes compared with control subjects and exercise increased adipose tissue GLUT4 protein expression by 36 %. Skeletal muscle GLUT4 protein expression did not differ in control subjects and diabetes patients.

A study by McGee et al. (2009[68]) shows that a single bout of exercise is sufficient to induce the GLUT4 protein expressions in human skeletal muscle and GLUT4 enhancer factor (GEF) and myocyte enhancer factor (MEF)2 transcription factors are required for this response. Another study by Gurley et al. (2016[31]) indicated that 4 weeks of voluntary wheel running (VWR) increased the GLUT4 protein expression in obese mice through increased Glut4 transcription. It is reported that Glut4 gene transcription elevates by 1.8-fold and GLUT4 mRNA level rises 2 to 2.5 fold within 3 hours after a single bout of exercise in both human and rat skeletal muscle (Ren et al., 1994[87]; Neufer and Dohm, 1993[78]; Krajewski, 2000[53]; Garcia-Roves et al., 2003[23]). Exercise increases GLUT4 protein expression and insulin sensitivity in parallel. Two hours of swimming per day for 5 days increased GLUT4 protein concentration and insulin sensitivity by 87 % and 85 %, respectively, in comparison to controls. GLUT4 protein concentration and insulin sensitivity were still higher by 52 % and 51 %, respectively after 24 hours of training (Kump and Booth, 2005[58]). Impaired expression and translocation of GLUT4 in muscle cells are the main reason for insulin resistance and type 2 diabetes. Exercise increases GLUT4 content and GLUT4 translocation to the plasma membrane in type 2 diabetes, thereby increasing glucose transport and insulin sensitivity. Therefore, exercise can be an important modality for the treatment of type 2 diabetes.

### Regulation of mitochondrial biogenesis by exercise

Regular exercise induces mitochondrial biogenesis, resulting in increased lipid oxidation capacity and turnover and improved glucose transport (Bruce et al., 2006[8]). Mitochondrial biogenesis is the process by which new mitochondria are produced, increased capacities for respiration, oxidation, and energy expenditure (Holloszy and Booth, 1976[37]). As such, mitochondrial dysfunction is associated with insulin resistance in skeletal muscle, which may lead to type 2 diabetes and obesity (Befroy et al., 2007[5]; Krssak et al., 1999[56]; Mogensen et al., 2007[75]; Toledo et al., 2007[99]). 

Fewer and smaller-sized mitochondria are found in skeletal muscle of insulin-resistant, obese, or type 2 diabetes subjects, and the size of mitochondria correlates with mitochondrial oxidative capacity (Morino et al., 2005[76]; Kelley et al., 2002[46]; Ritov et al., 2005[88]). Impairment of mitochondrial function leads to a reduction in the volume of lipids targeted for oxidation, resulting in an accumulation of fatty acids and their metabolites (Lowell and Shulman, 2005[62]). Increased intracellular levels of these lipids' metabolites are functionally linked to impaired insulin sensitivity in skeletal muscle. Mitochondrial dysfunction may trigger lipid droplet formation by switching metabolic pathways to glycolysis and fatty acid biosynthesis (Lee et al., 2013[61]). A study by Morino et al. (2005[76]) reported that the reduction of mitochondrial content that results from a sedentary lifestyle also causes intramuscular lipid accumulation, defects in insulin signaling, insulin resistance, and, ultimately, type 2 diabetes. Obesity can also result in mitochondrial deregulation through alterations in crucial transcriptional activators such as PGC-1 as well as impaired fusion and fission, leading to aberrant mitochondrial morphology. These changes can subsequently lead to reduced oxidative capacity and cause lipid metabolite accumulation, increased oxidative stress, and production of reactive oxygen species (ROS). Collectively, these studies show that mitochondrial dysfunction leads to impaired insulin signaling pathways and increases the risk of type 2 diabetes.

Studies show that vigorous aerobic exercise results in an increase of mitochondrial DNA (mtDNA) as well as in oxidative capacity (Hollozy, 1967[36], 2004[35]; Hood, 2001[38]; Hood et al., 2006[39]). Proportionality between mtDNA content and its oxidative capacity in skeletal muscle has been determined, prompting the “gene dosage” theory that postulates mtDNA replication is an integral mechanism for exercise-induced mitochondrial biogenesis (Williams et al., 1986[105], 1987[104]). Studies conducted on animals show a proportional increase of citrate synthase (CS) activity and mtDNA by exercise (Hood, 2001[38]; Williams et al., 1987[104]). Besides, in studies conducted in healthy lean individuals, the activity of CS was shown to correlate with mtDNA content in vastus lateralis muscle (Wang et al., 2000[103]; Freyssenet et al., 2004[22]). It is reported that athletes generally have higher mtDNA content in muscle than sedentary individuals and mtDNA content is proportional to mitochondrial volume density (Puntschart et al., 1995[84]). A study by Granata et al. (2016[27]) indicated that 4 weeks of sprint interval training in 29 healthy males resulted in an increase of mitochondrial volume density and the CS. Also, 6 weeks of moderate continuous intensity training in 21 healthy men showed an increase in mitochondrial volume density without influencing mitochondrial respiration (Meinild Lundby et al., 2018[72]). These studies are indicating that exercise increases mitochondrial volume density in both animals and humans. 

Exercise induces higher steady-state mitochondria content and an increase of dependence on the duration, frequency, and intensity of performed exercise (Hickson et al., 1981[34]; Dudley et al., 1987[17]; Hood et al., 2000[40]). A study by Toledo et al. (2007[99]) shows that combined physical activity and a weight loss program increase the mitochondrial size and density. Studies conducted on younger, lean adults who habitually perform high-intensity exercise show that these individuals are associated with an increased capacity for fat oxidation (Goodpaster et al., 2003[25]; Menshikova et al., 2005[73]). In an experiment with men and women aged 21-87 years, Vittone et al. (2003[101]) show that 16 weeks of aerobic physical activity increases the activity of citrate synthase and cytochrome c oxidase by 46 % and 76 %, respectively.

During exercise, there is an increase in several molecular 'stress' signals in skeletal muscle that appear to be responsible for the initial activation of mitochondrial biogenesis after exercise. These molecular signals include elevated levels of cytosolic Ca^2+^, AMP, and reactive oxygen species (ROS) (McConell et al., 2010[66]; Ojuka et al., 2003[81]; Irrcher et al., 2003[43]). Increasing cytosolic Ca^2+^ levels in L6 muscle cells via caffeine treatment activates Ca^2+^/calmodulin kinase (CAMK) and increases markers of mitochondrial biogenesis, which include PGC-1, mitochondrial transcriptional factor a (Tfam), and citrate synthase (McConell et al., 2010[66]; Ojuka et al., 2003[81]). Activation of muscle AMPK by 5'aminoimidazole-4-carboxyamide-ribonucleoside (AICAR) in L6 muscle cells also increases many of these mitochondrial biogenesis markers (McConell et al., 2010[66]). Increasing ROS levels in skeletal muscle cells activate the redox sensitive kinases AMPK and p38 MAPK and results in elevated PGC-1; these ROS effects were blocked by co-treatment with antioxidants (Irrcher et al., 2003[43]). Increased mitochondrial content by exercise protects against insulin resistance and type 2 diabetes by increasing the capacity of mitochondria to oxidize fats and their derivatives.

### Mechanism of Calmodulin-dependent protein kinase (CaMK)II activation in skeletal muscle

**Figure 2:**
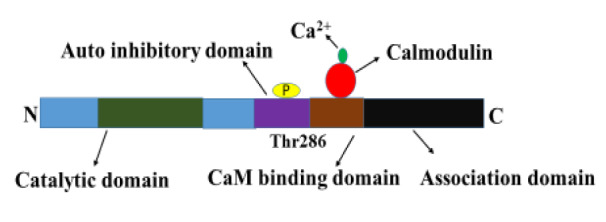
Structure of active CaMKII. Calcium/calmodulin-dependent protein kinase II consists of a catalytic domain, an autoinhibitory domain and an association domain. Binding of calmodulin to CaM binding domain results in a conformational change in CaMKII that exposes the catalytic domain and enables the Thr286 to be phosphorylated.

CaMKII is a multi-functional Ca^2+^/Calmodulin-dependent serine/threonine-specific protein kinase (Nghiem et al., 1993[79]). It activates when cytosolic Ca^2+^ levels rise. CaMKII is a multimeric holoenzyme composed of 8-12 units and each subunit has a catalytic, autoinhibitory, and association domain (Figure 2(Fig. 2)) (Shen and Meyer, 1998[94]; Hudmon and Schulman, 2002[42]). The amino terminus catalytic domain includes ATP and substrate binding sites; it is responsible for the transfer of phosphate from ATP to serine or threonine residues in substrates. The autoinhibitory domain contains a CaM-binding domain, and it features a pseudosubstrate site, which binds to the catalytic domain and prevents its ability to phosphorylate proteins (Kanaseki et al., 1991[45]). The carboxyl terminus amino acid association domain is necessary for the formation and assembly of CaMKII holoenzyme.

CaMKII is different from other CaM kinases owing to its ability to autophosphorylate at Thr286. Binding of Ca2+/CaM to the CaM-binding domain of CaMK activates enzymes through their structural arrangement into subunits that expose Thr286 in the autoinhibitory domain and the catalytic domain (Payne et al., 1988[82]). A typical individuality of CaMKII is that upon activation by CaM binding, kinase undergoes phosphorylation at Thr286 amino acid residue, making kinase partially unassimilated with Ca^2+^/CaM (Hudmon & Schulman, 2002[42]). When Thr286 residue is phosphorylated, it successfully blocks autoinhibition, allowing for permanent activation of the CaMKII enzyme and gaining Ca^2+^/CaM independent activity. The CaMKII remains to activate until it is dephosphorylated by phosphatase (Colbran et al., 1989[11]; Hanson et al., 1994[33]).

Studies show that CaMKII can be activated in human skeletal muscle by exercise (Rose et al., 2006[91]) and in rats, fast-twitch muscle by in situ electrical stimulation (Rose et al., 2007[90]). A study by Egan et al. (2010[19]) shows that high-intensity (80 % VO_2_ max) cycling exercise increases phosphorylation of CaMKII at Thr286. Another study by Serpiello et al. (2011[93]) also shows that phosphorylation of CaMKII increased by 69 % after an acute sprint exercise in young adults. Calcium-independent CaMKII activity was increased by 47 % in muscle after 7 days of stretch overload and after 2 weeks of voluntary wheel running. Nevertheless, there was no increase in calcium-dependent or total CaMKII activity, indicating that there was greater activation of pre-existing CaMKII (Fluck et al., 2000[21]). Besides, a study performed by Joseph et al. (2018[44]) indicated that 5 bouts of 17 minutes swimming exercise for 5 days increases phosphorylation of CaMKII at Thr286 in rat skeletal muscle. These studies confirm that exercise increases phosphorylation of CaMKII in both animals and humans.

### Role of CaMKII in GLUT4 expression, mitochondrial biogenesis, and histone modification 

Exercise induces several molecular signals in skeletal muscle, which are responsible for the initial activation of mitochondrial biogenesis after exercise. These molecular signals include increased levels of cytosolic Ca^2+^, AMP, and ROS (Irrcher et al., 2003[43]; McConell et al., 2010[66]; Ojuka et al., 2003[81]). Increased cytosolic Ca^2+^ levels in L6 myotubes through caffeine treatment activates Ca^2+^/Calmodulin kinase and increases markers of mitochondrial biogenesis, which include PGC-1α, Tfam, COX, and citrate synthase. Conversely, inhibition of CAMKII activity completely prevents Ca^2+^-induced increase of PGC-1α expression and mitochondrial biogenesis (Ojuka et al., 2003[81] McConell et al., 2010[66]). PGC-1 is a transcriptional co-activator that interacts with a broad range of transcription factors that are involved in a wide variety of biological responses, including adaptive thermogenesis, mitochondrial biogenesis, and glucose/fatty acid metabolism. Raising cytosolic Ca^2+^ in epitrochlearis muscle induces an increase in PGC-1 expression and mitochondrial biogenesis and this adaptation is prevented by inhibiting CaMKII. 

A study by Mukwevho et al. (2008[77]) reported that caffeine-induced CaMKII activation increases the binding of MEF2A to Glut4 promoters and GLUT4 expression in C2C12 myotubes. CaMKII-induced expression was diminished by KN93 treatments. MEF2 is a transcriptional activator; it relies on recruitment and co-operation with other transcription factors to drive the expression of its target genes. Another study by Smith et al. (2007[96]) showed that high-intensity exercise resulted in increases of autonomous CaMKII activity, increases in MEF2A-bound Glut4, and increases in GLUT4 mRNA and protein expression in the rat triceps muscle. 

Chromatin comprises genomic DNA wrapped around a core of histone proteins (Strahl and Allis, 2000[98]). The spatial relationship between DNA and the histone core determines the transcriptional status of surrounding genes. A study by McKinsey et al. (2001[71]) reported that a tight association between DNA and histones results in transcriptional repression and the loose association between DNA and histones results in increased transcriptional activation. Furthermore, associations between DNA and histones are controlled by post-translational modifications such as acetylation, phosphorylation, and methylation of histones proteins (Strahl and Allis, 2000[98]). For example, acetylation induces the removal of positive charge from histones and decreases the association between histones with the negatively charged phosphate group of DNAs. As a result, tight electrostatic interaction between histones and DNA converts to a more relaxed structure that is associated with greater levels of gene transcription (Strahl and Allis, 2000[98]). Acetylation is closely associated with increases in transcriptional activation, while deacetylation is linked with transcriptional deactivation. Mechanisms for acetylation and deacetylation take place on the NH_3_^+^ groups of lysine amino acid residues. Acetylation is regulated by a factor called Histone acetyltransferases (HATs). HATs help the transfer of an acetyl group from a molecule of acetyl Coenzyme-A to the NH_3_^+^ group on lysine. Deacetylation of lysine is facilitated by a factor called Histone deacetylases (HDACs), which catalyzes the removal of the acetyl group with a molecule of H_2_O (Kuo and Allis, 1998[59]; Grunstein, 1997[30]).

**Figure 3:**
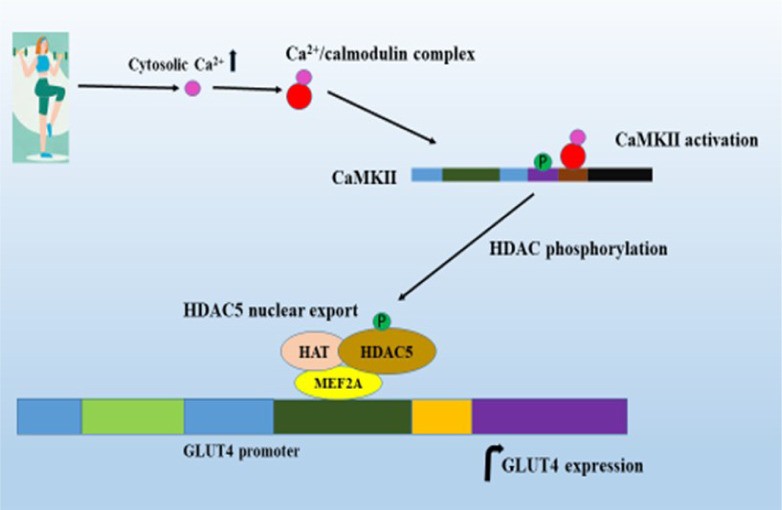
CaMKII activation by exercise increases GLUT4 expression. Exercise activates the binding of Ca^2+^/calmodulin complex to the CaM binding domain, resulting in the phosphorylation of Thr286 that activates CaMKII. CaMKII activation causes export of HDAC resulting in increased MEF2 gene transcription; and MEF2 together with HAT and other transcriptional factors increase the GLUT4 expression.

A study by McGee and Hargreaves (2004[69]) showed that a single bout of exercise reduces the amount of HDAC5 in the nucleus of human skeletal muscle. In addition, over-expression of HDAC5 attenuated the adaptations to exercise training in mouse skeletal muscle (Potthoff et al., 2007[83]). Exercise exports HDAC from DNA, thereby removing their transcriptional repressive function. CaMKII activation by exercise exports nuclear HDAC from DNA by phosphorylation (Grozinger and Schreiber, 2000[29]; McKinsey et al., 2000[70]; Lu et al., 2000[63]; Wang et al., 2000[103]; Backs et al., 2006[3]). A study by Smith et al. (2007[96]) reported two-fold increases of CaMKII phosphorylation in Wistar rats after they completed 5 x 17 min bouts swimming session. Exercise-induced CaMKII phosphorylation diminished when KN93 was administrated prior to exercise. CaMKII activation exports HDAC from DNA by phosphorylation, which may increase the accessibility of MEF2 to their binding domains and allow the recruitment of co-activators such as PGC-1 to stimulate the expression of target genes (Grozinger and Schreiber, 2000[29]). Export of HDAC favors increased HAT activity. Associations of HDAC free MEF2 and HAT together with co-activator molecules such as PGC-1 facilitate the acetylation of MEF2. This increases the rate of binding of MEF2 to the transcriptional activator and results in increased GLUT4 gene expression (Figure 3(Fig. 3)).
